# “I felt like a little kind of jolt of energy in my chest”: embodiment in learning in continuing professional development for general practitioners

**DOI:** 10.1007/s10459-024-10332-4

**Published:** 2024-04-29

**Authors:** Stense Kromann Vestergaard, Torsten Risor

**Affiliations:** 1 Section of Education, Steno Diabetes Center Copenhagen, Borgmester Ib Juuls Vej 83, Herlev, Copenhagen 2730 Denmark; 2 Section of General Practice, Department of Public Health, University of Copenhagen, København, Denmark; 3 Department of Community Medicine, Faculty of Health Sciences, UiT The Arctic University of Norway, Tromsø, Norway

**Keywords:** Embodiment, Learning, General practitioners, Continuing professional development, Micro-phenomenology

## Abstract

Learning in medical education encompasses a broad spectrum of learning theories, and an embodiment perspective has recently begun to emerge in continuing professional development (CPD) for health professionals. However, empirical research into the experience of embodiment in learning in CPD is sparse, particularly in the practice of general medicine. In this study, we aimed to explore general practitioners’ (GPs’) learning experiences during CPD from an embodiment perspective, studying the appearance of elements of embodiment—the body, actions, emotions, cognition, and interactions with the surroundings and others—to build an explanatory structure of embodiment in learning. We drew on the concepts of embodied affectivity and mutual incorporation to frame our understanding of embodiment. Four Danish and three Canadian GPs were interviewed to gain insight into specific learning experiences; the interviews and the analysis were inspired by micro-phenomenology, augmented with a complex adaptive systems approach. We constructed an explanatory structure of learning with two entrance points (disharmony and mundanity), an eight-component learning phase, and an ending phase with two exit points (harmony and continuing imbalance). All components of the learning phase—community, pride, validation, rehearsal, do-ability, mind-space, ambiance, and preparing for the future—shared features of embodied affectivity and mutual incorporation and interacted in multi-directional and non-linear ways. We discuss integrating the embodiment perspective into existing learning theories and argue that CPD for GPs would benefit from doing so.

## Introduction

Learning in medical education encompasses a broad spectrum of theories from cognitive perspectives through experiential and situated learning to reflective practice (Dong et al., 2021; Kaufman & Mann, 2010; Mann & Sergeant, 2013). Generally, the development of continuing professional development (CPD) for general practitioners and family physicians (collectively referred here to as GPs) has been based on pragmatic and need-based incentives, with learning theory playing only a minor role (Brown et al., 2019; Grierson & Vanstone, 2021). As is true more broadly for CPD for health professionals, theories that have been suggested and applied are frequently cognitive and outcome-based (Bednar et al., 2021; Moore et al., 2009; Ramani et al., 2019). However, more recently, the use of embodied learning theories in formal health professional education (HPE) has been an area of exploration (Kinsella, 2015; Loftus & Kinsella, 2021).

The embodiment perspective in HPE, encompassing CPD, signals a paradigm shift in the conceptualization of learning. This shift is characterized by a transition towards a more comprehensive understanding of learning that incorporates sensory, emotional, and interactional dimensions, alongside the current emphasis on cognition and skills. Such a transformative change holds significant implications for the future methodologies and advancements in CPD. Pioneering work has advanced embodiment in CPD for health professionals by exploring it as a theoretical foundation of learning (Dieckmann et al., 2017; Fernandez, 2020; Kelly et al., 2019; Snoeren et al., 2015). Theories introduced in this literature draw from phenomenology inspired by Merleau-Ponty (Merleau-Ponty, 2012), Shilling’s body pedagogics (Schilling, 2017), installation theory by Lahlou (Lahlou, 2017) and Varela, Thompson and Rosch’s enactivism (Varela et al., 1991) but no single definition stands out as the go-to in the field of CPD for health professionals. Most of the related literature comprises conceptual papers, and empirical inquiries into GPs’ experiences of embodiment in learning are scarce indeed. This scarcity invites for investigation into experiences of learning in CPD in order to develop the understanding of the structure and concept of the embodiment perspective in this field.

In a qualitative interview study based on a postgraduate program, Jaye (2004) found that GPs assign various meanings to the concept of embodiment, including an implicit holism across the various dimensions of daily life, complex relationships between body, soul, and mind, and the social meanings of the body. While these findings provide insight into how GPs’ interpret the concept of embodiment, they shed no light on their lived experiences of embodiment in learning. The learning experiences of GPs could be essential for further developing the perspective of embodiment in learning and for building a learning theory that encompasses this perspective. Our study aim was two-fold: (1) to contribute to the growing understanding of embodiment in learning in CPD by exploring the experiences of GPs and (2) to identify an explanatory structure of embodiment in learning in CPD for GPs.

### Conceptualizing embodiment

While interest in embodiment in CPD for health professionals is increasing, little agreement exists about its interpretation and application at a conceptual level. In the absence of shared understandings and definitions, we offer our conceptualization of embodiment as the basis for the study. Our understanding of embodiment is inspired by phenomenology and social cognition (Fuchs, 2016; Merleau-Ponty, 2012). This understanding is adopted from general perspectives on embodiment not specifically evolving around HPE or CPD.

The body, our primary way of perceiving the world, is a unity; our various body systems, i.e., visceral, tactile, and motor, are inseparable (Merleau-Ponty, 2012). Merleau-Ponty (2012) described our entanglement with the world as individual sensory and historical beings: “I am everything that I see and I am an intersubjective field, not in spite of my body and my historical situation, but rather by being this body and this situation and being, through them, everything else” (p. 478). In the body, every experience is entangled. Different modalities, such as vision and touch, are experienced in unity, rather than being translated from one modality to another (Merleau-Ponty, 2012). This entanglement means that emotions are not simply mental phenomena; we experience them through bodily sensations (Fuchs, 2016). Bodily resonance—all specific and general body sensations—and the emotional affordances in the environment interact dynamically as embodied affectivity. This perspective suggests that during CPD, health professionals engage in learning that involves the utilization of all their senses, even if one or several senses are primarily targeted by the learning method. For instance, when a GP attends a lecture, the primary focus of the learning method may be auditory, supplemented by visual aids such as slide presentations with a focus of enhancing knowledge. However, alongside these sensory experiences, the GP will concurrently undergo emotional responses and other bodily sensations in dynamic interactions throughout the session.

The dynamic interactions also include intentions to act; that is, bodily resonance can pull us toward or away from something, someone, or a situation (Fuchs, 2016; Tanaka, 2015). Fuchs (2016) describes these cycles of interactions or feedback:

Being affected by the affective affordances or value features of the situation (‘affection’, impression) triggers a specific bodily resonance which in turn influences the emotional perception of the situation and implies a corresponding expression and action readiness (‘e-motion’). Embodied affectivity consists in the whole interactive cycle which is crucially mediated by the resonance of the feeling body. (p. 197)

Thus, the body and its surroundings are intimately related; as Merleau-Ponty (2012) notes, “(t)o be a body is to be tied to a certain world” (p. 149). Part of embodiment in the world is the relationship to objects and others in the surroundings; i.e., embodied inter-affectivity. Emotions are perceived and expressed between subjects as gestures and actions, kinetics and intensity (Fuchs, 2016). Social cognition arises from the “moment-to-moment interaction of two subjects” (Fuchs & De Jaegher, 2009, p. 466), and intersubjectivity consists of “entering a process of embodied interaction and generating common meaning through it” (Fuchs & De Jaegher, 2009, p. 465). Mutual incorporation occurs as the lived bodies of both subjects extend and form a common incorporeality, and Tanaka (2015) explains that,” (i)ntercorporeality contains a perception-action loop between the self and the other” (p. 462). It includes embodied inter-affectivity as emotions are perceived and expressed between subjects.

Applying this perspective to CPD suggests that the GP attending the aforementioned lecture, while already immersed in the entanglement of various senses, would also undergo shared interactions with both the lecturer and fellow attendees. This interaction would foster a reciprocal loop of perception and incentive to act, evolving through engagement with others. The collective experience in the CPD setting becomes a dynamic process where mutual incorporation with the lecturer and peers creates a feedback loop that is part of the overall learning experience.

In this study, the complex interweaving of bodily sensations, emotions, and cognitions and impressions and expression in continuous interaction with others and the surroundings is the perspective from which we view and understand learning.

### Aim

The study aim was to explore GPs’ experiences of learning during CPD from an embodiment perspective. We wanted to explore the appearance of the elements of embodiment: bodily sensations, emotions, reflection, cognition, actions, and interactions with surroundings, including people, artifacts, and milieus. Further, we aimed to explore the dynamic relationships between elements to build an explanatory structure of embodiment in learning for GPs in CPD.

## Method

### Reflexivity

This research was conducted by a junior researcher, SKV, with a background in public health and skilled in qualitative research methodologies. SKV’s experience includes involvement in the development of CPD programs and health professional education initiatives. This research endeavor was undertaken in collaboration with and under the guidance of a senior qualitative researcher TR, with a background as GP as well as anthropology, coupled with extensive experience in both the development and research realms of health professional education. This collaborative partnership between SKV and TR has fostered a sense of curiosity within the research framework, as it avoids the constraints typically associated with individuals from similar disciplinary backgrounds. However, it has also confined the research within the established boundaries of HPE, CPD, and learning. SKV took the lead in conducting interviews, transcribing data, and conducting initial analyses, with assistance provided by TR. The results, interpretations, and any deviations from the established methodology were subject to thorough discussion and mutual agreement within the collaborative framework. It is noteworthy to acknowledge that SKV’s lack of medical training may have influenced the dynamics of the interview interactions. On one hand, interviewees may have felt compelled to provide explanations for circumstances that would have otherwise been tacit assumptions or shared knowledge among peers with similar professional background. Conversely, interviewees might have omitted certain explanations, deeming them too intricate or complex to convey to someone not initiated into the general context of general practice. To mitigate this potential bias, the adoption of the MP method was intended and assumed to facilitate a more comprehensive dialogue, transcending mere theoretical understanding.

### Context, setting and design

The primary CPD setting was clinic-based facilitated small group learning following the model of practice-based small group learning (PBSGL) developed in Canada (Kelly et al., 2007) and further refined in Scotland (Armson et al., 2007; Cunningham & Zlotos, 2016, 2019; Kelly et al., 2007). This group learning approach has been implemented in many countries, including Denmark (Kompetencecenter, 2017). PBSGL is a systematic mono-professional atheoretical approach that consists of problem-, evidence- and dialog-based facilitated learning sessions building on written materials, including cases (Zaher & Ratnapalan, 2012). In 2021, a clinic-based version was introduced in Denmark. The fundamental principles of written material that includes cases and a facilitated dialog session are the same as in the original PBSGL, but this Danish version takes place in clinic settings and includes all professions; i.e., GPs, nurses, secretaries, midwives and others (Decentral Gruppebaseret Efteruddannelse, n.d.). The objective is for participants to acquire knowledge and reach a shared agreement about how to approach specific disease treatments or management approaches that can be implemented in the clinic (Læger.dk, n.d.). We chose clinic-based PBSGLs because they are conducted in person and interprofessional, incorporating both facts and dialogue. We anticipated that in this setting, if learning occurred it could incorporate embodied affectivity and mutual incorporation by encompassing both cognition, sensory, emotional, and interactional dimensions.

As we also wanted to explore how embodiment in learning in CPD for GPs varies different cultural and pedagogical contexts, we included learning experiences among GPs in CPD settings in Montreal, Quebec, Canada. This allowed us to study potential differences and similarities between two nationalities and learning in different settings under different learning methods. The study is designed to explore and offer a broad understanding of an embodiment perspective in CPD learning not as a comparative study.

### Participants and data collection

Two groups of interviewees were recruited. Danish GPs who had participated in the clinic-based PBSGL formed a group of homogeneous learning activities with a focus on human interaction and Canadian GPs whom forming a group of heterogeneous with learning activities. These variations between these groups in both learning activities and cultural context allowed us to begin to explore if and how these factors influenced the embodiment perspective in learning.

In March-October 2022, SKV conducted seven interviews with Danish and Canadian GPs who had participated in CPD activities. Danish interviewees were sampled from GP clinics in Denmark that had conducted clinic-based PBSGL within the previous year and recruited by emailed invitations from the CPD organization responsible for administering the clinic-based PBSGL. All 26 GPs that had participated in a clinic-based PBSGL from June to December 2021 was invited in retrospective. From January to June 2022 invitations were included in communication to the 12 facilitators from the CPD organization responsible for administering the clinic-based PBSGL. Four GPs volunteered to be interviewed. Canadian interviewees were convenience sampled from GPs in Montreal who had participated in CPD during the previous year and recruited through a GP network at the Institute of Health Sciences Education (IHSE) at McGill University. Three GPs volunteered to be interviewed. They had participated in an online version of PGSGL, a conference workshop, and a web-based individual learning activity, respectively. Five interviews were conducted in person and two were conducted online; each interview lasted 40–65 min and was audio recorded.

### Micro-phenomenology

Embodied inter-affectivity is a constant state, but not all emotions, sensations, and cognitions or the dynamic connections between them rise to the level of conscious awareness (Fuchs, 2016). Thus, we anticipated that past experiences would leave both mental memories and embodied traces in conscious and pre-reflective forms. Micro-phenomenology (MP) is an interview method that supports subjects in accessing their subjective embodied experiences by re-evoking an experience to the degree where it becomes almost more present for the subject than the current moment (Petitmengin, 2006). Using MP allowed us to help GPs re-evoke embodied experiences of learning and the entangled elements of bodily sensations, emotions, reflection, cognition, actions, and interactions.

One of the strengths of micro-phenomenology is its potential to turn attention away from the *what* and towards the *how* (Petitmengin, 2006). By using this approach, we focused on how GPs experienced learning, not what they learned. The interviewer asked each GP to choose a specific moment of learning during a CPD experience and supported each interviewee in re-evoking the experience, assisting them to:

…rediscover the spatio-temporal context of the experience (when, where, with whom), and the precision of the visual, auditive, tactile and kinesthetic, olfactory and possibly gustatory sensations associated with the experience, until the past situation is ’re-lived’, to the point that it is more present than the interview situation. (Petitmengin, 2006 p. 244–245)

The interviewer maintained a non-inductive attitude. An attitude characterized by content-free inquiries refrains from prompting, suggesting, or provoking specific content or answers (Petitmengin, 2006) Instead, she sought to support interviewees in re-establishing contact with the experience, a state that was revealed through verbal clues like the use of ‘I’ statements, specific time and place indicators, non-verbal clues like an unfocused, wandering gaze, and para-verbal clues like slowing of speech and unconscious use of gestures (Petitmengin, 2006).

### Analysis

**Fig. 1:**
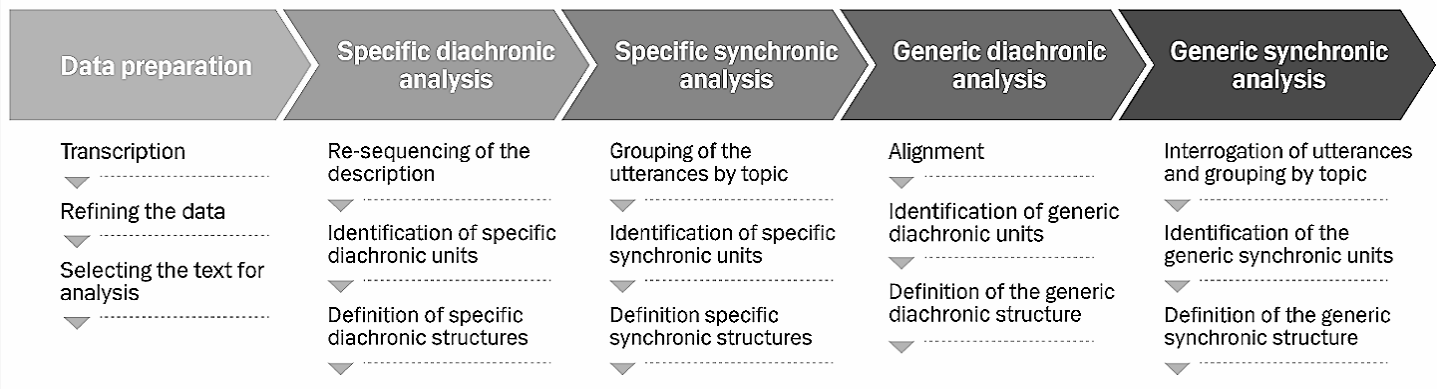
Micro-phenomenology analyses procedure. Five steps with each three stages. Illustration adapted from Fig. 1. In Valenzuela-Moguillansky and Vásquez-Rosati (2019)

The analysis followed the five steps described by Valenzuela-Moguillansky and Vásquez-Rosati (2019) (Fig. 1).

#### Data preparation

SKV listened to the audio-recorded interviews and transcribed them verbatim in their original language. All interview transcriptions were read in full, and the utterances from evocation states were selected for analysis. SKV first removed general utterances about experiences, then utterances related to satellite dimensions of experiences (Valenzuela-Moguillansky & Vásquez-Rosati, 2019). Satellite dimensions include technical and theoretical knowledge; judgments like evaluations, commentaries, and beliefs; and goals, including intentions and motives (Petitmengin et al., 2019; Valenzuela-Moguillansky & Vásquez-Rosati, 2019). Utterances about the context of experiences were retained only to inform our understanding of the settings in which they occurred.

#### Specific analysis: diachronic and synchronic units and structure

A central part of the analysis was identifying units of meaning also referred to as *descriptemes* in MP (Petitmengin et al., 2019). These being diachronic units describing temporal sequences of the experience and synchronic units describing details of the content of the learning experience (Petitmengin et al., 2019). Units are the building blocks af the analysis and examples will be integrated in the results section. They take the form of short quotes, often just a few words and sometimes sentences.

First, utterances from each interview were re-sequenced to construct a chronological timeline. SKV then sorted units into moments and organized them into a diachronic structure comprising phases and sub-phases these were analyzed to establish criteria and names for each..

Guided by our aim of exploring the elements of embodied learning, SKV then identified synchronic units that comprised different combinations of the elements; emotions, cognition, action, sensations and interactions with others, i.e., individuals or groups and specific or more general people such as a colleague or a community. After identifying subphases based on meaning units in each learning experiences, a synchronic structure for each interview was established (Valenzuela-Moguillansky & Vásquez-Rosati, 2019). These structures were represented in dynamic lines showing the diachronic structure horizontally with a timeline evolving from left to right and the synchronic structure unfolding from top to bottom (Valenzuela-Moguillansky & Vásquez-Rosati, 2019).

#### Example

To illustrate this in a simplified version we take the example of Steven a Canadian GP: First, we describe four diachronic phases, representing the temporal development of the learning experience. Then, we delve into one of these phases to explore an example of the synchronic dimensions.

Steven is sitting in his office by the computer, engaging in online CPD, a routine he follows every day—a mundane situation (diachronic phase 1). Suddenly, he realizes that what he is reading is relevant to him (diachronic phase 2). Subsequently, he starts saving the material, preparing for the future (diachronic phase 3). Finally, he reaffirms in his mind the applicability of what he has just learned, signifying the end of the learning experience (diachronic phase 4). In the diachronic phase 2, Steven experiences sensations of various modalities. These are the synchronic units. He feels a pleasant surprise—a sense of a short jolt of energy in his chest. He enjoys the feeling; it feels good. Almost simultaneously, he experiences a cognitive sensation of intention—he wants to know more. He desires to act and explore more literature on the subject.

#### Generic analysis: diachronic and synchronic units and structure

The generic analysis was conducted in two steps. First, all data from Danish interviews were analyzed together to create a generic structure. SKV first aligned the specific structures of the four Danish interviews to identify generic diachronic units, based on the external timeframe defined by the start of the learning experiences (Valenzuela-Moguillansky & Vásquez-Rosati, 2019). SKV then ordered these units to form a diachronic structure through an iterative interrogation process, followed by ordering synchronic units through an alternating top-down and bottom-up analysis. We then defined a generic structure of embodied learning in CPD for GPs.

**Fig. 2:**
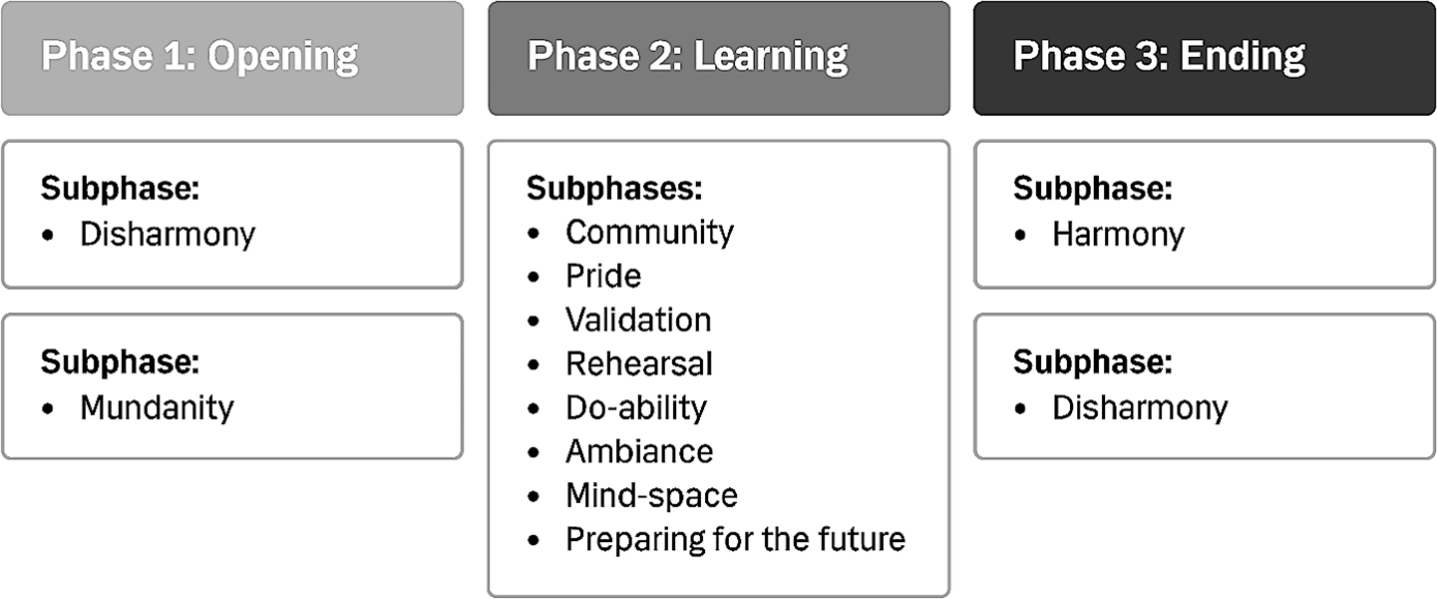
Generic structure of the learning experience

Secondly, specific structures from the Canadian interviews were added one at a time to identify components of the generic structure that were or were not reinforced and any new components that emerged. Finally, a shared generic structure was created to illustrate the learning experiences of all interview participants (Fig. 2).

In recognition of the many layers of experiences in the learning phase and acknowledgment that synchronic units could not always be ordered linearly as subphases, we applied the complex adaptive system (CAS) theory described by Paley and Eva (2011) to refine our analytical perspective. CAS refers to a system composed of many interacting components or agents that adapt and evolve based on their interactions. These systems are characterized by their dynamic and nonlinear nature, meaning that small changes can lead to significant and unpredictable outcomes. CAS can help to explain phenomena that are not easily understood by examining individual components in isolation (Paley & Eva, 2011). CAS was applied to assist us in building an explanatory structure of embodied learning (Paley, 2010; Paley & Eva, 2011). In this study, we applied CAS to a specific phase of the learning experience— the learning phase. By approaching the eight subphases, identified in the learning phase, as components in a complex adaptive system we were able to generate a dynamic structure of the interactions between the components. Each component identified in the generic analysis had connections to other components. Analyzing and mapping these ‘rules’ of connection creates a structure that is different from the diachronic-synchronic matrix of MP, but still with explanatory qualities.

### Ethics

Approval from the Central Denmark Region Committee on Health Research Ethics was not required according to the Consolidation Act on Research Ethics Review of Health Research Projects. A data management plan for the Danish part of the study was approved by the Capital Region of Denmark (VD-2018-303). The Steno Diabetes Center Copenhagen and the Copenhagen University Department of Public Health approved the project. The Canadian part of the study was approved as an amendment to the original project by the Institutional Review Board at McGill University (IRB Internal Study Number: A07-E25-22B). Interviewees signed an informed consent form or provided verbal consent when interviewed online. They were assured about confidentiality and their anonymization in interview transcriptions. The names figuring in this paper are substitute names given by the authors.

## Findings

**Fig. 3:**
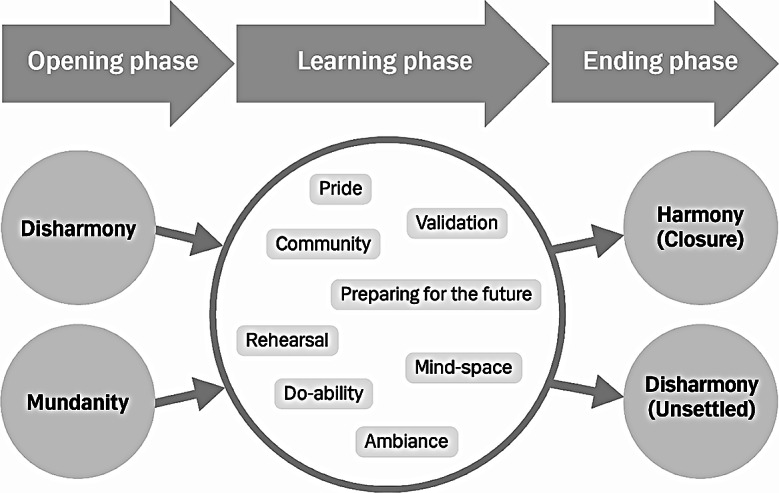
Final generic structure of the learning experience in CPD for GPs

The final structure of embodiment in learning for GPs had three phases: (1) an opening phase with two different ways of entering the learning experience, (2) a central and more complex learning phase, and (3) an ending phase that ended in different two ways (Fig. 3). GPs entered the learning phase from a sense of either disharmony or mundanity. The experiences of all four Danish participants began from a sense of disharmony; i.e., some kind of disturbance or imbalance (Fig. 3). When the Canadian data were added, it became evident that the GPs in Montreal had a different starting point of more mundane learning situations. These were characterized by less tension and a more everyday approach to the situations in which the learning occurred. The central learning phase comprised eight components: community, pride, validation, rehearsal, mind-space, ambiance, do-ability, and preparing for the future (Fig. 3). Data from interviews with Danish GPs revealed two end points: one characterized by closure and the re-establishment of harmony and the other characterized by being less settled and continuing disharmony. These also corresponded to the experiences of Canadian GPs (Fig. 3).

### The opening phase: disharmony or mundanity

In the disharmony variation of the opening phase, learning was experienced as evolving from disjuncture and imbalance. It materialized as worry about the future (Sofia), a discrepancy between practice and guidelines (Peter), a recurring problem in everyday practice (Mia), or a breakthrough in dealing with a specific problem in the future (Emma). Opening phases that were characterized by disharmony created opportunities for learning and changes related to a specific patient or group of patients, clinic-level collaboration, or the future of global health. Experiences of disharmony included various emotional states, e.g., fear about the future or irritation arising from feeling in doubt, and cognitive reflections on the discrepancies between clinical guidelines and the realities of daily clinical practice. These emotions and reflections induced curiosity that informants felt in their heads.

In the mundanity variation of the opening phase, GPs experienced a sudden realization that something would be useful in further practice because it addressed a need for updating materials (Steven), presented a new diagnostic model (Ann), or added new knowledge about patient management (Paul). These experiences were accompanied by the sense of a pleasant surprise, including a jolt of energy in the chest (Steven) and a brief moment of excitement (Paul).

### The ending phase: re-establishing harmony or continuing disharmony

Learning experiences culminated differently across interviewees. Most interviewees experienced some kind of closure or conclusion; i.e., a re-establishment of harmony. They had created plans and prepared for moving toward the future and were fueled by feelings of contentment and engagement. Some interviewees also anticipated difficulties in implementing new practices but did so in the context of an overall feeling of confidence expressed as “we can do it” (Sofia), “we can make a change and make an impact” (Emma) or “will use this, it is relevant” (Mia).

Other GPs experienced continuing imbalance at the end of the learning experience, including unabated disharmony and uncertainty about whether recommended practices could or should be applied. This left the GPs in states of discouragement or doubt – a kind of continuing or ongoing disharmony.

### The learning phase: a complex structure of embodiment

The learning phase was composed of eight components that were interrelated through interactions and mechanisms and formed a complex structure of embodiment.

#### Community

Experiences of community were about relationships to others and comprised the elements of cognition, actions, sensations, emotions and the presence of others, imagination and action incentive. Community was experienced as other group members nodded and smiled, participated, and did not hold back in interactions or dialog. As Emma said, “(E)hh, I can definitely feel a different energy in the room… people contribute, pay attention, nod, are present… they are not doing other things. They participate in the discussion, they look around at each other, they smile.” These gestures constituted a shared sense of belonging to a community. The circular interplay of the constituents of community) creates the impression and expression of mutual incorporation. The experience of community fueled learning in the sense that it was experienced as a positive thing, i.e., feelings of shared direction and goals, being able to plan actions for the future, and doing things right. It included feelings like calm, comfort, confidence and trust and was described as a warm and comfortable feeling situated around the throat and neck or as the feeling of something tingling in the abdomen. Sofia described it this way: “… (I)t gives me a feeling of community, that’s always nice… it is a warm feeling, it is placed somewhere around here… [gesturing with her hands behind her neck]… around the neck and throat, something pleasant.”

Alternatively, the experience of community was described a feeling that provided motivation, similar to adrenaline before a running race (Sofia), or as the action resulting from a shared decision, e.g., “(W)e do what we are told and leave the worries behind” (Mia).

A specific aspect of cognition was a sense of shared mental or cognitive movement, a recognition of shared incentive and agreement to take action an element we named action-incentive, this was only experienced in in-person activities. Another aspect of cognition was imagination, imagination of others, this aspect was present when learning activities were not extensively based on interactions. These two aspects created two different cycles of mutual incorporation. A cycle of mutual incorporation based on present others and a cycle based on absent other which we named: imagined mutual incorporation.

#### Pride

Only Danish GPs experienced the component of pride, which was a combination of emotions, actions and the others in the room. It was recognized by individuals as an interplay between emotions and bodily sensations, similar to intercorporal resonance. GP’s feelings were expressed in actions of leaning forward and looking around and impressions of others’ actions—smiles, nods, and leaning forward—all resulted in a sense of shared pride. As Mia described it, “It is a feeling of *yes*, now we have something new, we can contribute. I feel relaxed and happy.”

#### Validation

Only Canadian interviewees experienced validation. It consisted of a combination of cognition, emotions, and the other in the form of the larger GP community. It was experienced by individual GPs as a recognition that their practices were right, according to the prescribed procedures. Steven said, “(I)t is more like okay… ok (I’m) not crazy, this is good… I guess I can keep on… it was more about reassuring and confirming. Helping me to understand that I was, you know, behaving and acting properly in my clinical practice.” This gave rise to positive emotions of confidence and happiness and gave GPs a sense of belonging to the professional GP community.

#### Rehearsal

Rehearsal was composed of action, cognition, sensations, and the other as present in person or through imagination. Actions were very central to this component of the learning. When GPs were with others, actions were negotiated and ways of addressing patients were rehearsed (Peter and Sofia). When they were alone, rehearsal was cognitive through imagining a specific patient and testing and memorizing actions; Paul said, “…(T)esting different things thinking of her, as well I guess, you know, would this medication work for her.”

One GP saw herself in short film clips that played in front of her eyes and head (Ann). The version of rehearsal that involved others showed several aspects of mutual incorporation, although emotions did not play a great part. The version with absent others involved imagination and mental movements. For those rehearsing with absent others, the use of imagination and the experience of a mental movement constituted a different feedback loop. This loop was internal, similar to embodied affectivity but with a very clear presence of imagination.

#### Mind-space

Interviewees explained how their experiences could not be situated either inside or outside of their heads but were located in a different space that was simultaneously both internal and external. The mind-space connected the inside and outside and was experienced as a combination of imagination, sensations, and surroundings. Some GPs experienced a brightness that was both in the room around them and inside them, part of the lightness of the situation (Peter and Emma). For Ann, another GP, her mind’s activity was not inside her head; it was in a bigger space surrounding her, which she called the brain space: “Well, I don’t think it was ever just inside. Well, I think it’s like in my mind but you know when we are looking at ideas, we are looking around us also, right?… So I think that it’s not like in my brain, because that will feel very small. So it is around just outside but it is kind of part of my brain space.” The component of mind-space introduced an understanding of how GPs perceived their experiences of learning.

#### Ambiance

Only interviewees in in-person learning settings experienced the component of ambiance. It was an example of mutual incorporation with a strong focus on the surroundings, other people, and the space between people, combined with emotions and sensations. Ambiance was related to learning with others and to being in the room with others, sensing the mood and atmosphere outside oneself and feeling relaxed and comfortable inside and experiencing others as being open, happy, proud and free in their movement (Emma). Another attribute of ambiance was feeling safe and being able to volunteer for a demonstration at a conference workshop without experiencing it as being on a stage; Ann said, “I didn’t feel like I was like you know on a stage or anything like that.” Ambiance connected the surroundings of the atmosphere in the room with individuals’ internal positive experiences and enhanced their ability to engage and feel comfortable with those around them.

#### Do-ability

Another commonly shared component of the learning experience was do-ability. This component comprised emotions, sensations, action, the other, cognition and, for some GPs, imagination. The common experience of this component was considering the applicability of new knowledge, skills or guidelines. One GP described this as experiencing a skill she just learned as do-able in the sense manageable or actionable. For two GPs, this consideration was related to doubt about whether the guideline could and should be implemented. Peter said,… It fills me with a little bit of discouragement. I think, well, yes it says so in the instruction, but what do I do with the fact that it is probably not possible to implement, even if I try. And do I have the energy at all to try.” One GP’s experience was particularly sensation-based, as she described something nagging or spiky inside, experienced as a kind of brief discomfort or disturbance behind her head (Mia).

For other GPs, the component of do-ability was more pleasant because they experienced implementation as do-able. As Ann said, “…(O)h, you know, I could see… bringing this into the clinic, I could see doing this with a patient… having this skill will give more weight to what I do, like imagining things, like how you would incorporate it.” For one GP (Paul), the experience was of collaborating with others. Part of the positive experience was the sensation that ‘we can do it’ experienced by GPs in the body as energy, like adrenaline, that motivated and created the incentive to act. For one GP (Ann), the experience was also pleasant and occurred largely through mental images as she was able to visualize herself performing the skills. While realizing it was doable, she experienced sensations of warmth and lightness both in and around her body and the emotional sensations of happiness, engagement and confidence. Simultaneously, she experienced an absence of the body, i.e., not noticing and not minding whether her chair was comfortable. Indeed, evaluating do-ability was a significant part of the learning experience and was experienced as interactions between different sensory modalities and entanglement that resembled the loop of mutual incorporation but with a twist. In addition to other people who were physically present, the other was also the sense of a medical community, a whole healthcare system, or an imagined patient.

#### Preparing for the future

Preparing for the future involved many actions – producing action plans, retrieving material, cutting, pasting, and saving documents, and placing materials so they were easy to access. Paul noted that, “(T)here was a table with the treatments and I thought, well, I can take out this whole table and I could put it on the wall but then I would never read it. And then I noticed there was a QR code leading you to the same information and you know an easy access from the phone.” The experience also involved cognitive activity in the form of planning and negotiating with oneself or with others. This activity was encouraged by the feeling of engagement and intention to make use of the new knowledge, skills and agreements. Preparing with others demonstrated mutual incorporation and intercorporal resonance, while preparing by oneself primarily dealt with interactions with the surroundings by placing prints and documents in easily accessible places.

### Interactions between components in the learning phase

The general structure of the learning phase comprised many interactions between its eight components. The components were related through the different elements of action, emotions and cognition, including imagination, and the others and the surroundings in a complex adaptive system. Some, like rehearsal and preparing for the future, were primarily action-centered, while others, like community and ambiance, were directed at the context, including people. Still others, like pride and validation, were mostly centered on emotions. Two components stood out from the rest due to their unique qualities. Do-ability took the form of a question interviewees asked themselves, and mind-space explained a specific way of interpreting information during the learning phase.

The main differences between components depended on whether the learning experience was with other people or individual. In-person situations resulted in mutual incorporation, whereas individual experiences involved more imagination. In addition, the component of pride was only experienced by Danish GPs in group-based meetings and validation was only experienced by Canadian GPs in more individual learning situations; this difference may arise from differences between settings, cultural contexts, or both.

In analyzing interactions between components in the learning phase with the aim of describing a structure, we discovered that they could not be understood in terms of directionality and had no starting or ending point. All components were related to at least one other component, and they collectively formed the interconnected structure of learning phase experiences.

#### Tracing the interactions and building a dynamic structure

**Fig. 4:**
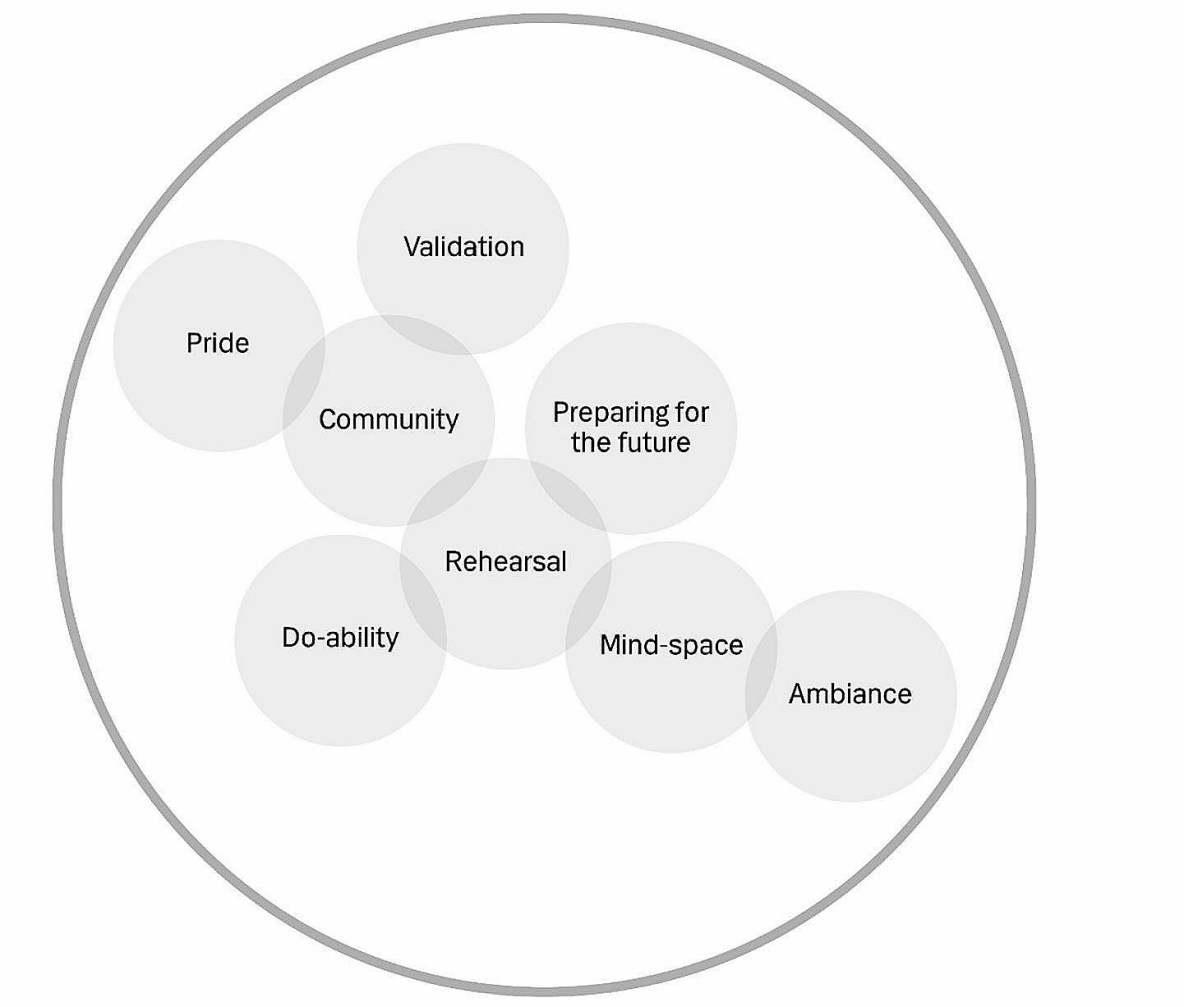
The interconnectedness of the eight components of the complex adaptive system of the learning phase

We began to develop the structure from the community component because many interviewees described it as a significant and strong part of the learning experience (Fig. 4). The sense of being a part of and belonging to a community was closely related to both the emotion-driven components of pride and validation and the action-related component of rehearsal. Pride was only experienced by Danish GPs who were physically present in a group and the element of expressions/impressions in the form of nodding and smiling was shared by the pride and community components. Validation was experienced among Canadian GPs in more individualized settings, but the sense of belonging to a community connected validation to the community component (Fig. 4).

The relationship between community and rehearsal was established through real or imagined actions of practicing together. Also related to imagination was the experience of mind-space. Imagination and sensation took place both inside and outside the head and body, and mind-space dissolved the barriers between internal and external experiences. Although GPs experienced the component of mind-space individually, the component of ambiance was only experienced by GPs attending in-person PBSGL events. Ambiance connected the individual with the surroundings based on mutual incorporation, which has a very different quality than the dissolution of barriers between internal and external experiences of mind-space (Fig. 4).

Do-ability was also connected to rehearsal and community and experienced differently depending on whether interviewee’s learning experiences were in-person or individual. The experience of in-person situations resembled mutual incorporation, whereas individual experiences comprised imagination. In-person experiences were related to the component of community through the sense of being able to manage something or produce a shared plan. The interaction of do-ability with the component of rehearsal was established through a shared focus on action. Rehearsal is also related to preparing for the future through a shared focus on action. The final structure (Fig. 3) includes the dynamic of the three phases and the complexity of interactions in the learning phase (Fig. 4).

## Discussion

### Mutual incorporation and embodiment

The findings of this study suggest a structure of embodiment in learning in CPD for GPs,

consisting of three distinct phases. Each of these phases; opening to learning, learning, and ending learning, is further delineated into subphases. The opening phase encompasses two subphases: ‘mundane’ and ‘disharmony,’ representing the varied entry points of interviewees into their learning experiences. Similarly, the concluding phase of the learning experiences is segmented into subphases: ‘harmony’ and ‘disharmony,’ reflecting the interviewees’ experiences of either closure or unsettlement as the learning experiences end. In the learning phase, the structure of embodiment in learning for GPs in CPD comprised the eight components of community, pride, validation, rehearsal, mind-space, ambiance and do-ability. Each component included the elements of actions, emotions, sensations, cognitions, and relations to others and the surroundings to varying degrees. The components were inter-related through shared elements, allowing the creation of a structure of embodiment in learning. The presence of these elements indicated that all the components of the learning experience were embodied and reflect the concepts of embodied affectivity and mutual incorporation; i.e., the experience of a loop between emotions, sensations, cognitions, and impressions of others (Fuchs, 2016).

Ideally, the envisioned structure would have outlined a singular trajectory through the learning experience—a single entry, a sequential progression through the components of the learning phase, and a singular exit from the learning experience. However, the analysis revealed that such a linear and unidirectional framework did not align with the data in this study. Specifically, the learning phase was identified as a complex entity characterized by multifaceted and multidirectional dynamics. The intricate nature of the learning phase suggested that interviewees’ experiences involved layered and diverse interactions within the learning context.

### Imagined mutual incorporation

Interestingly, the experience of learning when alone or in a less interactive space often involved imagination. Some scholars in the field of embodied cognition propose that imagination plays a part in learning (Alibali & Nathan, 2018): “In our view, imagining involves mentally experiencing actions by engaging in motor imagery or mental simulation of action” (p. 79). Imagination activates areas of the brain similar to those the actions themselves would have activated (Alibali & Nathan, 2018). We found that the imagination activated embodied affectivity similarly to the way that engaging with other humans did, in a kind of imagined mutual incorporation. The link between affection and imagination is not a new discovery. According to Fletcher (2021), imagination “work(s) as a mediator between reason and understanding” (p. 3), “invoke(s) our memory and orient(s) our reasoning” (p. 2) and “couple(s) meanings from our previous experiences with our current lived circumstances” (p. 4). A special feature of things imagined is that they are not true but are still experienced as real (Fletcher, 2021). It is also worth noting that in the component of mind-space, GPs described imagination as not only experienced inside the head; imagination and bodily sensations merged, removing the barrier between internal and external experiences and creating an imagination-based embodied affectivity and mutual incorporation, sometimes with imagined others, sometimes with the imagined self.

### What learning theory adds

Learning in CPD, medical education, and in general has been viewed from different theoretical perspectives including cognitive, experiential, and cultural-social approaches, and reflective learning, among others. We briefly visit these perspectives to interrogate how they align with and add meaning to our findings.

#### Cognitive learning

We have already seen the role that imagination plays in an embodied cognitive approach to learning (Alibali & Nathan, 2018). The cognitive perspective on the learning process is based on ideas of receiving, organizing, storing, and retrieving information (Dong et al., 2021). From this perspective, learning is a mental process centered in the mind, and the learner is active and responsible in the process (Dong et al., 2021; Kaufman & Mann, 2010).

The component of preparing for the future relates to both cognitive learning and imagination. Imagined in the sense that all the scenarios and cases were imagined. Cognitive in the sense of figuring out what to do in the future by discussing options and selecting actions and approaches. Approaches similar to what is suggested in many decision-making models. In the Danish context, preparing for the future was closely related to the sence of community and was facilitated through the mutual incorporation. Thoughts and planning were interwoven with interactions, intersubjectivity, emotions, and sensations.

#### Experiential learning

Another learning perspective with a substantial role in medical education is experiential learning. It focuses on the experiences of direct encounters that guide activities like apprenticeship, clerkship, and residency (Dong et al., 2021; Kaufman & Mann, 2010). With inspiration from Kolb et al. (2001), learning is about the actual situation, grasping it through the senses in immediate experience or by conceptualization and symbolic representation The learning experiences in this study did not involve experiences of direct patient encounters, and simulation was limited to demonstration and practice in the conference workshop. During PBSGL, patients are represented as exemplary cases. Clearly, the senses still played a major part in all the components of the learning experience. Based on our findings, we could argue that experience and the senses are important not only in the practice of skills in workplace learning and simulation, they are also at the core of any part of learning even without direct focus on integrating the body. Our findings show that learning is experienced through the body and the senses, not just for instrumental purposes but as part of embodied affective dynamics and in mutual incorporation.

#### Communities of practice and situated learning

Sociocultural perspectives on learning augment the cognitive and experiential learning perspectives that focus on individuals. In the perspective of situated learning (Lave & Wenger, 1991; Wenger, 2011), learning occurs through learners’ participation in activities in a community of practice. Learning in communities of practice plays out in the interaction between the people (the community), the skills, knowledge, and behavior (the practices), and the field of general practice (domain) (Dong et al., 2021; Kaufman & Mann, 2010). Participation is essential to learning in communities of practice, but learning can be both formal and informal. In the situated learning perspective, learning is inseparable from the situations in which it takes place and, because knowledge is socially constructed and context dependent, learning occurs in relationships between people rather than in an individual’s head (Kaufman & Mann, 2010).

The situated learning perspective helps explain many of the components of learning experiences we identified. Learning happened in situations and in relation to others, either in person or as a conceptual and imagined community. This is evident in the components of community, pride, and validation. In the components of preparing for the future and rehearsal, the co-construction of knowledge is explicit. However, a situated learning perspective sheds little light on the role of emotions and bodily sensations in learning.

#### Reflective learning

Reflective learning is an individual-based learning perspective that takes emotions into account (Dong et al., 2021; Kaufman & Mann, 2010). In the concepts of reflection-in-action or reflection-on-action (Schon, 1983), experience, including its emotional aspects, is central to a cycle of looking at experiences while or after they happen (Dong et al., 2021). Reflection includes introducing cognitive activities to evaluate the experiences and enable changed actions in the future (Dong et al., 2021). These forms of reflection are evident in the components of do-ability, rehearsal, and preparing for the future. However, we also found that GPs recognized these emotions happens through their bodies and sensations. Kinsella (Kinsella, 2008) has introduced an embodied perspective on reflective practice and suggested how this interpretation could be beneficial in educational settings.

In sum, these learning perspectives provide insight into cognition, including imagination, experience through the senses, social interaction, and reflection. However, they all omit the ever-present physicality in and through which all these learning perspectives are interrelated: the body, with its inherent possibilities and limitations, that constitutes the physical structure of embodiment. We cannot think or reflect separately from the body or experience any phenomena except through the body. When we interact in communities and social learning milieus, we do so with our own bodies and those of others.

This is not a call to dismiss learning theories that focus on cognition, experience, or communities, or reflection. Instead, we argue for broadening the perspective on learning in CPD for GPs to encompass the body through the embodiment perspective.

### Limitations

We aimed to explore learning in an embodied perspective, and our approach, inspired by MP, resulted in finely detailed descriptions of experiences. However, the final analytic step proved insufficient to deal with the complexity of the data, necessitating the use of CAS in the analysis. Valenzuela-Moguillansky andVásquez-Rosati (2019) presented an illustration of the refined structure of their exemplary MP analysis that included circular movement, suggesting that the linear nature of analysis is not always compatible with the data and complexity of the phenomena being studied. It would though be valuable to apply the MP analytic approach to a larger dataset to see if our challenges arose from a small dataset.

The predominant emphasis of the discussion revolved around the intricacies and dynamics of the learning phase, allocating minimal space for grappling with the interpretation and theoretical aspects inherent in the two variations of both entering and concluding the learning experience. This narrowed focus may have limited the exploration of broader conceptual and interpretative frameworks related to the diverse entry and exit experience. Future considerations might benefit from a more comprehensive examination of the theoretical underpinnings and interpretative nuances associated with the distinct phases of initiation and closure in the context of the learning experiences studied.

Although the Danish data were based on interprofessional learning activities, we did not take the experiences of other professionals into consideration. It would be beneficial to study embodiment in learning separately in mono- and interprofessional contexts to explore whether and how these contexts shape learning experiences in an embodied perspective.

### Future studies

This study is the first of its kind and relatively limited in scope and our findings are suggestive, not affirmative. Additional empirical studies of learning experiences in CPD among GPs would be of great value to gain a broader understanding of the dynamic structures, components, and elements described here. Including but not limited to whether the Danish interviewees’ experience of ‘pride’ was correlated with the specific opening phase of ‘disharmony’ they encountered. And similarly, if the unique component of embodied inter-affectivity, termed ‘validation,’ observed exclusively among Canadian GPs, could be associated with the opening phase of ‘mundanity’ they also experienced. Additional studies should also explore how embodied learning is experienced in different contexts, e.g., online, individual, and group-based, mono- and interprofessional, or using different learning methods, such as art-based, web-based, lectures, etc. It may also be of interest to extent the inquiry into the potential relevance for CPD in other medical specialties and for other health professionals.

## Conclusion

We identified a structure of an embodiment perspective in learning for GPs in CPD with three phases: opening, learning and ending. The learning phase comprised eight components in dynamic interaction with each other: community, pride, validation, rehearsal, do-ability, preparing for the future, ambiance and mind-space. Each component comprised emotions, cognitions, sensations, interactions, and, sometimes, imagination. We identified the dynamics as mutual incorporation. This intersubjectivity was experienced when learning took place in groups, however, imagining others supported a similar dynamic when learning was individual, which we refer to as *imagined mutual incorporation*. These components are not entirely new in learning theories but viewing them together along with a centrality of the body adds new perspective in understanding GPs’ learning experience in CPD. However, further studies providing more insight into the complex and dynamic interactions between these components of learning could add to the development of the structure of an embodiment perspective in learning. Incorporating an embodiment perspective into in our understanding of learning could potentially change the way we develop, conduct, and assess learning in CPD for GPs.
